# Supplementary material for: Bayesian refinement of protein structures and ensembles against SAXS data using molecular dynamics
Source: PLoS Comput Biol. 2017 Oct 18;13(10):e1005800. doi: 10.1371/journal.pcbi.1005800 (PMC5662244; doi:10.1371/journal.pcbi.1005800)
Supplement: S1 Archive — (BZ2) [file pcbi.1005800.s011.tar.bz2 › gromacs_4.62_bayesian_saxs_refi/share/html/online/g_sas.html]

g\_sas


| g\_sas Main Table of Contents |

**VERSION 4.6  
Sat 19 Jan 2013**

---

### Description

g\_sas computes hydrophobic, hydrophilic and total solvent
accessible surface area. See Eisenhaber F, Lijnzaad P, Argos P,
Sander C, & Scharf M (1995) J. Comput. Chem. 16, 273-284.
As a side effect, the Connolly surface can be generated as well in
a .pdb file where the nodes are represented as atoms and the
vertice connecting the nearest nodes as CONECT records.
The program will ask for a group for the surface calculation
and a group for the output. The calculation group should always
consists of all the non-solvent atoms in the system.
The output group can be the whole or part of the calculation group.
The average and standard deviation of the area over the trajectory can be plotted
per residue and atom as well (options -or and -oa).
In combination with the latter option an .itp file can be
generated (option -i)
which can be used to restrain surface atoms.

By default, periodic boundary conditions are taken into account,
this can be turned off using the -nopbc option.

With the -tv option the total volume and density of the
molecule can be computed.
Please consider whether the normal probe radius is appropriate
in this case or whether you would rather use e.g. 0. It is good
to keep in mind that the results for volume and density are very
approximate. For example, in ice Ih, one can easily fit water molecules in the
pores which would yield a volume that is too low, and surface area and density
that are both too high.

### Files

| option | filename | type | description |
| --- | --- | --- | --- |
| **-f** | traj.xtc | Input | Trajectory: xtc trr trj gro g96 pdb cpt |
| **-s** | topol.tpr | Input | Structure+mass(db): tpr tpb tpa gro g96 pdb |
| **-o** | area.xvg | Output | xvgr/xmgr file |
| **-or** | resarea.xvg | Output, Opt. | xvgr/xmgr file |
| **-oa** | atomarea.xvg | Output, Opt. | xvgr/xmgr file |
| **-tv** | volume.xvg | Output, Opt. | xvgr/xmgr file |
| **-q** | connelly.pdb | Output, Opt. | Protein data bank file |
| **-n** | index.ndx | Input, Opt. | Index file |
| **-i** | surfat.itp | Output, Opt. | Include file for topology |

### Other options

| option | type | default | description |
| --- | --- | --- | --- |
| **-[no]h** | bool | no | Print help info and quit || **-[no]version** | bool | no | Print version info and quit || **-nice** | int | 19 | Set the nicelevel || **-b** | time | 0 | First frame (ps) to read from trajectory || **-e** | time | 0 | Last frame (ps) to read from trajectory || **-dt** | time | 0 | Only use frame when t MOD dt = first time (ps) || **-[no]w** | bool | no | View output .xvg, .xpm, .eps and .pdb files || **-xvg** | enum | xmgrace | xvg plot formatting: xmgrace, xmgr or none || **-probe** | real | 0.14 | Radius of the solvent probe (nm) || **-ndots** | int | 24 | Number of dots per sphere, more dots means more accuracy || **-qmax** | real | 0.2 | The maximum charge (e, absolute value) of a hydrophobic atom || **-[no]f\_index** | bool | no | Determine from a group in the index file what are the hydrophobic atoms rather than from the charge || **-minarea** | real | 0.5 | The minimum area (nm^2) to count an atom as a surface atom when writing a position restraint file (see help) || **-[no]pbc** | bool | yes | Take periodicity into account || **-[no]prot** | bool | yes | Output the protein to the Connelly .pdb file too || **-dgs** | real | 0 | Default value for solvation free energy per area (kJ/mol/nm^2) |

---

http://www.gromacs.org  
gromacs@gromacs.org
